# Adaptation of Proteasomes and Lysosomes to Cellular Environments

**DOI:** 10.3390/cells9102221

**Published:** 2020-10-01

**Authors:** Yohannes Afework Mebratu, Zerihun Hailemariam Negasi, Saugata Dutta, Joselyn Rojas-Quintero, Yohannes Tesfaigzi

**Affiliations:** Pulmonary and Critical Care Medicine Division, Department of Medicine, Brigham and Women’s Hospital, Harvard Medical School, Boston, MA 01255, USA; ymebratu@bwh.harvard.edu (Y.A.M.); znegasi@bwh.harvard.edu (Z.H.N.); sdutta@bwh.harvard.edu (S.D.); jrojasquintero@bwh.harvard.edu (J.R.-Q.)

**Keywords:** protein degradation, core particle, regulatory particle, endosome, autophagy, aggresome

## Abstract

Protein degradation is important for proper cellular physiology as it removes malfunctioning proteins or can provide a source for energy. Proteasomes and lysosomes, through the regulatory particles or adaptor proteins, respectively, recognize proteins destined for degradation. These systems have developed mechanisms to allow adaptation to the everchanging environment of the cell. While the complex recognition of proteins to be degraded is somewhat understood, the mechanisms that help switch the proteasomal regulatory particles or lysosomal adaptor proteins to adjust to the changing landscape of degrons, during infections or inflammation, still need extensive exploration. Therefore, this review is focused on describing the protein degradation systems and the possible sensors that may trigger the rapid adaptation of the protein degradation machinery.

## 1. Background

Protein homeostasis or ”proteostasis”, the balance of the synthesis and degradation of proteins, is closely associated with cellular compartments, including the Golgi apparatus, the endoplasmic reticulum (ER), and the lysosome [1]. The ER is not only responsible for the synthesis of the protein backbone, but also for monitoring the quality of the newly synthesized proteins through the unfolded protein response (UPR) system and shuttles misfolded proteins into the degradation pathway. The ubiquitin-proteasome and the autophagy-lysosome systems ensure selective degradation of proteins. Depending on the cell type, the relative contribution of the proteasome and autophagy systems may vary considerably, but the current accepted estimate is that the proteasome is responsible for the great majority (up to 80%) of the protein degradation in the majority of growing mammalian cells. However, in muscle cells, for example, autophagy can account for 40% of degradation of long-lived proteins [2]. The crosstalk between these two proteolytic pathways increases the capacity to process protein degradation and participates in the recycling of the compounds [3]. Should the degradation of proteins be overwhelmed despite both degradation pathways being activated, unwanted proteins are sequestered into aggresomes to prevent toxicity to the cell.

This review will focus on the mechanisms that provide the plasticity of these degradation systems to adapt to physiological changes, including viral and bacterial infections or exposure to environmental insults that increase reactive oxygen species (ROS).

## 2. Proteasome Plasticity

**Figure 1:**
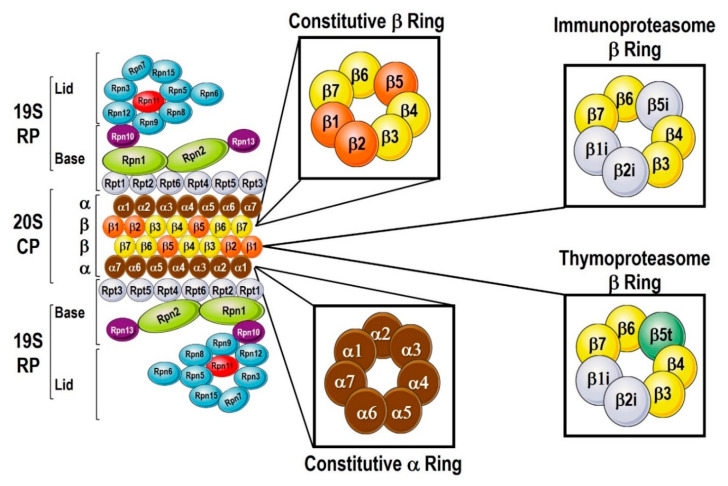
Proteasomal components. The proteasome can be divided into the 2 main structures: the 20S core protease (CP) and the 19S regulatory particle (RP). The 20S consists of α and β subunits, with 7 α-subunits forming the α-ring, and 7 β-subunits forming the β-ring. When the immunoproteasome is activated, there are 3 β-subunits that are replaced, β1i for β1, β2i for β2, and β i for β to form the Immunoproteasome. The CP in the thymus is equipped by β5t instead of the β5. The 19S RPs is a protein-complex made out 6 subunits of Rpt and 13 subunits of Rpn proteins important to keep the 20S CP open and to bind and process ubiquitylated proteins for degradation.

The term “proteasome” covers multiple types of entities that share a common proteolytic core, the catalytic 20S core particle (also called 20S CP). The 20S proteasome barrel is capped by different regulatory particles (RPs) or proteasome activators (PA) [4], including the19S, PA28α/β, PA28γ, or PA200 that can be on one or both sides of the barrel (Figure 1) [5]. At baseline, up to 75% of 20S CP remain uncapped, and the caps maintain the 20S core open and are crucial for binding and processing of the substrates [6]. Therefore, proteasomes should be viewed as being composed of building blocks that serve in the degradation of specific set(s) of substrates. This arrangement likely is crucial to cope with the diversity of the substrate types, the cellular compartments, and the speed by which proteins need to be processed.

The 20S Core Particle is a 700kDa protein complex [5], with a cylinder-like assembly that is made up of four axially stacked heptameric rings [5,6]. The two rings in the middle part consist of β subunits and are sandwiched between two α rings, ultimately forming a α_1-7,_β_1-7,_β_1-7,_α_1-7_ pattern (Figure 1). Seven similar but different subunits, α_1_ through α_7_, form the α ring and the *N*-termini of a subset of α subunits form the pore that controls the entry of incoming substrates. Several α subunits possess a nuclear localization signal and contribute to the subcellular localization of the proteasomes [7]. The β rings are made up of β_1-7_ subunits, but only the β_1_, β_2_ and β_5_ subunits show peptidylglutamyl-peptide-hydrolyzing, trypsin-like, and chymotrypsin-like catalytic activity, respectively [5,6]. Interestingly, individual overexpression of the catalytic subunits β5 and β1 is enough to increase the number of proteasomes in the cell.

19S Regulatory Particle consists of the Regulatory Particle of Triple-A ATPase (Rpt) subunits (Rpt1-Rpt6) and Regulatory Particle of Non-ATPase (Rpn) subunits (Rpn1-Rpn15) ranging 10-100 kDa in size, forming two sub-complexes, the lid and the base (Figure 1) [5]. The 19S particle Rpn1, Rpn10 and Rpn13 of the lid sub-complex act either directly [8]. via their own ubiquitin-binding domains (UBDs) or indirectly via the UBDs of shuttling proteins, such as p62 or Rad23 [9]. Deubiquitylation of incoming substrates is carried out by the deubiquitylating enzymes (DUBs) Rpn11, Uch37, and Ubp6/Usp14 [10]. The C-terminal domain of Rpn13 may also have deubiquitylating attributes, but the functions of the remaining lid subunits are largely unknown [6]. The Rpt subunits have a key role to provide energy for proteolysis, in an ATP-dependent process, to unfold the substrates and inject them into the 20S core. Further, Rpt2, Rpt3 and Rpt5 proteins possess conserved *C*-terminal motifs that allow insertion into the pockets between two contiguous α subunits of the 20S core particle [11]. Such insertion of Rpt subunits promotes α subunit rotation which facilitates gate opening and maintaining the open-gate conformation [5].

The lid and the base sub-complexes are formed independently before they are connected by the Rpn10 subunit [7]. Rpn14 (in mammals PAAF1 - proteasomal ATPase-associated factor 1) along with p28/gankyrin [12], 26S Proteasome Non-ATPase Regulatory Subunit 9 (also known as p27,PSMD9, bridge-1,RPN4), and S5b [13] serve as chaperones to facilitate the assembly of the 19S particle [14]. Such chaperones may also be important in the adaptation process of proteasomes.

**Figure 2:**
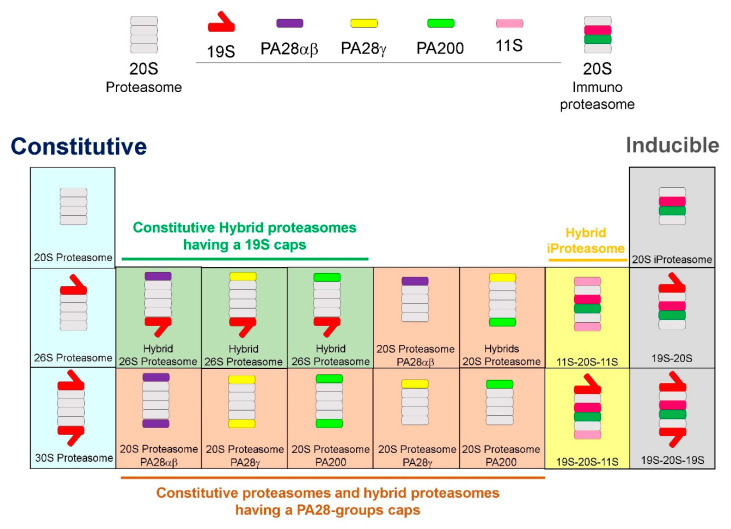
Types of Proteasomes. The constitutive proteasome is composed of the 20S CP and a single or double 19S cap to form the 26S or 30S proteasomes, respectively. When cells are exposed to inflammatory stimuli, i.e., TNF-α or IFN-γ, immunoproteasomes are assembled. The 20S CP incorporates βi-Ring, and is capped by PA28α/β, PA28γ, and PA200 caps on either one of both sides. Hybrid proteasomes have a 19S cap mixed with any of the activated caps (PA28α/β, PA28γ, and PA200). Hybrid iProteasome may be comprised of 11S, 20S, and 11S, or 19S cap, 20S, and 11S components. The inducible proteasome is composed of the 20S iProteasome and a single or double 19S cap to form the 19S-20S or 19S-20S-19S proteasomes, respectively.

The Protein activator (PA28) Regulatory or 11S Particle exists in two major forms, PA28α/β and PA28γ. While the constitutive proteasome is composed of the 20S CP and a single or double 19S cap to form the 26S and 30S proteasomes, respectively, when cells are exposed to inflammatory stimuli, such as TNF-α or IFN-γ, immunoproteasomes are assembled (Figure 2). When cells are exposed to IFN-γ, three genes, β1i, β2i, and β5i, (the *i* for inducible), replace the constitutive active subunits (β1, β2, and β5, respectively). In addition, INF-γ exposure also induces proteasome maturation protein (POMP) expression which facilitates assimilation of β1i, β2i and β5i into the 20S core particle. The 19S particle dissociates from the 20S CP [15,16] along with recruitment of PA28α/β RP in the cytosol or PA28γ in the nucleus, to form the “immunoproteasome” [15]. When PA200 is recruited, there is increased hydrolysis of peptide-sized substrates instead of larger intact proteins. Hybrid proteasomes can have a 19S and any of the activated regulatory particles, PA28α, PA28β, and PA200. In addition, iProteasome may be comprised of 11S, 20S, and 11S, or 19S cap, 20S, and 11S components (Figure 2). Hybrid immunoproteasomes, doubled-capped as PA28α/β-20S-19S, can form that are able to hydrolyze tri- and tetra-peptides more efficiently than the 26S proteasome [17]. The speedy assembly of immunoproteasomes [18], is crucial to process major histocompatibility complex (MHC) class I molecules during the early stages after infection [19]. However, the mechanisms that regulate the transition from one cap to another for the 20S CP are largely unknown. It is possible that different forms of the 20S proteasome coexist in the same cell, and even that a single 20S proteasome can be built of distinct α- or β-rings (Figure 2). In addition, different types of regulatory particles can be attached to the 20S CP either on one or both sides of the barrel (Figure 2).

A proteasome that is exclusively expressed in the thymus, designated as “thymoproteasome”, is found only in both human and mouse cortical thymic epithelial cells (cTECs) and has similar catalytic subunits as immunoproteasomes, except β5t instead of β5i. The β5t subunit is most closely related to β5 and β5i subunits while the other two catalytic subunits in the thymoproteasome are β1i and β2i, not β1 and β2. Thymoproteasome make up the majority of the proteasomes in cTECs, while the standard and immunoproteasomes are minimally represented [20].

Proteasomal components are heavily enriched in membrane-less organelles, also called proteasome storage granules (PSGs) [21]. As soon as the cell receives a signal of proliferation, such as glucose addition, PSGs disappear and proteasomes are repositioned into the nucleus. Therefore, proteasomes are located in perinuclear region during the G1 and early S phases and move to the periphery during G2 phase aided by cytoskeletal elements [22]. Up to 90% nuclear proteasomes can migrate to the cytoplasm the longer cells are in the quiescent state. RPN1 anchors proteasomes to the ER to form the endoplasmic reticulum-associated degradation (ERAD), less than 20 nm from the nucleus, whereas RPN9 anchors proteasomes to the nuclear pore complex (NPC) sites. Nuclear tethering of the proteasomes, for inner nuclear membrane-associated degradation (INMAD) [23,24], is mediated on two sites through RPN9 [21], on the or the nuclear core complex (NPC) via nuclear basket myosin-like protein (Mlp)—NPC interactome and directly via direct interaction with the Esc1p (Establishes silent chromatin 1p) [25].

## 3. Recognition of Proteins by the Proteasome

Proteins designated for degradation are tagged by ubiquitin, a protein composed of 76 amino acids. This tag is linked to the lysine residues of targeted proteins by 3 enzymes in an ATP-dependent process. Briefly, the E1 enzymes activate ubiquitin in an ATP-dependent manner and form E1-ubiquitin thioester. Then, the activated ubiquitin is transferred to E2 enzymes through an E2-ubiquitin thioester, Finally, an E3 ligase facilitates the formation of the isopeptide bond between the Lys of the substrate and the *C*-terminal tail of ubiquitin [26]. In the human proteome, there are only two E1, with 40 E2 ligases; however there are over 600 E3 ligases facilitating protein ubiquitylation (Table 1), suggesting that the specificity which protein undergoes ubiquitylation is determined by the E3 ligases [27]. The removal of the ubiquitin residue is regulated by six families of deubiquitinases (DUBs) (Table 1). Ubiquitin is recognized by 20 families of ubiquitin binding domains (UBDs), some of which are in RPN1, Rpn10 and Rpn13 [28]. The ubiquitin binding domains (UBDs) may differ in their affinity and avidity for distinct ubiquitin linkages, but their common feature is the single or multiple α-helices, zinc finger motifs, or pleckstrin-homology domains. Other UBD types show structural similarity to E2 conjugating enzymes without the catalytic activity UBDs can have ubiquitin-binding specificity, recognizing between the different ubiquitin tags. For example, BRAP (RNF52) preferentially binds to M1- and K63-linked di-ubiquitin chains, weakly to K27-linked chains, but not to K6-, K11-, or K48-linked chains [29]. The presence of several UBDs within the same protein not only increases the affinity to polyubiquitin chains, but can also change the avidity and determines linkage selectivity [30].

The proteasome can also bind polyubiquitylated substrates through proteins with ubiquitin like (UBL) domains that transiently associate with the 19S [31]. The best-studied UBL motifs are those interacting with SUMO, known as SUMO-interacting motifs (SIMs) and autophagy-related protein ATG8/LC3.

However, proteins containing unstructured regions, including p53, p73*α*, p21^Cip1^ I*κ*B*α*, MCL-1 and BIM, can be degraded in an Ub-independent manner by 20S proteasomes in vitro [32,33]. Also, ubiquitin-independent proteasomal degradations have been described for a wide range of proteins that possess unstructured peptide domains located within proteins for recognition by the proteasome [34,35]. For example, IκBα is constitutively degraded by the proteasome through a ubiquitin-independent mechanism [36]. Additionally, treatment of cells with dicoumarol, an inhibitor of NAD(P)H quinone oxidoreductase 1, induces p53 degradation by a proteasome-dependent, ubiquitin-independent mechanism [32].

## 4. Lysosomal Degradation and Recognition of Ubiquitylated Proteins

Lysosomes arise from the fusion of late endosomes that contain material taken up at the cell surface with transport vesicles that bud from the trans-Golgi network [37]. The lysosome can exhibit multiple tridimensional conformations, is highly dynamic, and is considered as a signaling hub because of its many possible functions [38]. The complex function stems partly from the 7–10 nm thick membrane that harbors 120 membrane-bound proteins not only maintaining intraluminal Na^+^, K^+^, Cl^−^, Ca^2+^ ion concentrations and pH (pH~5) [39], but also selectively mobilizing and accepting cargo by for example, the Lysosome Associated Membrane Proteins (LAMP)-1-3 and Lysosomal Integral Membrane Protein (LIMP-2). Encapsulated within the lysosome are ~60 acid hydrolases (e.g., glycosidases, proteases, lipases, nucleases, phosphatases, and sulfatases) that are responsible for the degradation of nucleic acids, proteins, lipids and carbohydrates [40]. Lysosomes are generally localized in the peri-nuclear region that is facilitated by E3 ligase RiNg Finger protein-26 (RNF26) controlling the microtubule-dependent transport of early and late endosomes [41]. In neuronal cells, transport of lysosomes is controlled by dynein motor proteins, that are associated with intermediate, light-intermediate, and dynein light chains [42]. The number of endolysosomal organelles as well as their fission and fusion in the perinuclear area is controlled by the ER through a complex system of tethering proteins [43].

Just as the proteasomal system is crucial for the normal turnover of cellular proteins, basal autophagy is crucial for intracellular quality control to selectively dispose of aberrant protein aggregates and damaged organelles. Aggresomes are confined by vimentin fibers and are enriched in the microtubule-organizing center (MTOC), attract chaperones and proteasomes, and serve as protein quality-control centers. Terminally aggregated proteins accumulate in an insoluble protein deposit (IPOD) and these IPODs exhibit a low rate of molecular exchange with the cytosol and are highly immobile [44]. These aggregates can induce autophagosome formation as they are large enough to allow simultaneous binding of multiple receptors to its surface.

In addition, some cell surface and integral membrane proteins [45], internalized ligands, larger protein aggregates including proteasomes, cellular organelles such as mitochondria, intracellular parasites, such as bacteria are degraded by the autophagy-lysosome pathway (Table 2) [46].

## 5. Ubiquitin-Like Proteins and Adaptors for Selective Autophagy

Autophagy requires the ubiquitin-like (UBL) protein, Atg12, to be conjugated to a conserved Lys residue of Atg5 and Atg16 [47] and together with an E2-like enzyme, Atg3, catalyzes the transfer of the activated microtubule-associated protein 1 light chain 3 beta (MAP1LC3B [48]. The lipidated LC3 (often referred to as LC3-II) allows for substrate uptake upon binding to several autophagy receptors [49]. This complex regulates closure of the phagophore by interacting with Cdc48/p97. [50], and by recruiting selective adaptors described below, directs specific targets to the lysosome for degradation [51,52].

**Figure 3:**
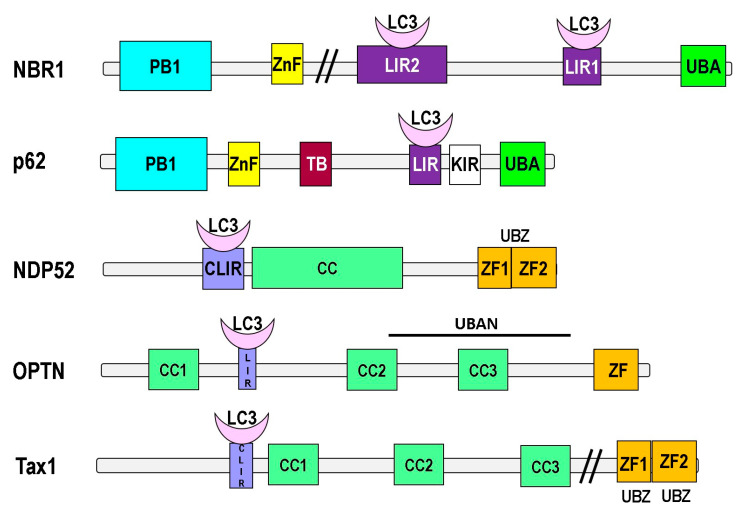
Autophagy adaptors. Ubiquitin recognition receptors are multiple in nature, but they share common areas that serve to recognize and bind to ubiquitin, and areas that allow recruitment of LC3 for the autophagophore. The receptors depicted are Neighbor of BRCA1 Gene 1 (NBR1), Ubiquitin-Binding Protein P62 (p62), nuclear dot protein 52; also known as calcium binding and coiled-coil domain 2 [CALCOCO2] (NDP52), optineurin (OPTN), Tax1-binding protein 1 (Tax1), Toll-Interacting Protein (TOLLIP), and NIX/BNIP3L [BCL2/adenovirus E1B 19 kDa interacting protein 3-like] (NIX/BNIP3). The ubiquitin binding domains are: ubiquitin-associated domain (UBA), Ubiquitin binding in ABIN and NEMO domain (UBAN), Ubiquitin-binding zinc finger domain (UBZ), Endoplasmic reticulum-associated degradation domain (Cue). The LC3 recruiting domains are: LC3-interacting region (LIR), non-canonical LIR motif (CLIR), and LC3 recognition sequence (LRS).

The sequestosome 1 (p62/SQSTM1), the first described mammalian autophagy receptor, is involved in regulating reactive oxygen species (ROS), apoptosis, inflammation, and autophagy [53,54,55]. It consists of an N-terminal Phex and Bem1p (PB1) domain [56], a zinc finger (ZnF) domain, Light Chain 3 (LC3) interacting region (LIR) [53,57], TRAF6-binding (TB), and ubiquitin-associated (UBA) domains [55] (Figure 3). The Keap-interacting region (KIR) interacts with Keap1 and affects the transcription factor Nrf2 and thereby controls ROS level [58]. The PB1 motif allows p62 to interact with itself and other PB1 domain-containing proteins and thereby hetero-, homo-, or polymerize. It is assumed that p62 first self-oligomerizes via its PB1 domain and thereby initiates aggregate formation of polyubiquitylated proteins, because individual p62 units poorly interact with ubiquitin [59]. In certain conditions, aggregates containing p62 and ubiquitylated proteins may even serve as a nucleating scaffold for autophagosome biogenesis, potentially by associating with mTORC1 and multiple Atg8/LC3 proteins [59,60]. The growing double membrane starts to enclose the p62-containing aggregates due to interactions between p62, Atg8/LC3, and other Atg proteins [61].

Neighbor of BRCA1 gene 1 (NBR1) is another adaptor protein with remarkable similarity to p62 (Figure 3). However, NBR1 is larger than p62, and its dimerization is mediated by a coiled-coil domain. Through its PB1 domain NBR1 interacts with p62 [62], but can also bind ubiquitin and LC3/GABARAP independently of p62, mediating autophagosomal degradation of ubiquitylated targets. Similar to p62, NBR1 accumulates in inclusion bodies upon autophagy inhibition and is required for the crosslinking of ubiquitylated proteins and aggrephagy [63]. Knockdown and overexpression experiments have shown that NBR1 is a major autophagy receptor for peroxisomes [64].

Optineurin (OPTN) is an adaptor protein with roles in signal transduction, membrane trafficking, and cell division. Optineurin possesses several coiled-coil domains that mediate its oligomerization, a LIR, and C-terminal UBAN and UBZ domains (Figure 3). Optineurin interacts with misfolded proteins in both ubiquitin-dependent and -independent fashion to mediate aggrephagy. TBK1 binds and phosphorylates optineurin within its LIR motif (Ser177), thereby enhancing its interaction with LC3/GABARAPs and potentiating target clearance [65]. Especially, the LIR of optineurin is required for aggrephagy and xenophagy [66], to restrict intracellular pathogens [65]. Increased expression is linked to glaucoma and amyotrophic lateral sclerosis (ALS) and is frequently found in pathological protein inclusions (reviewed in [67]).

The Calcium binding and Coiled-Coil Domain 2 (CALCOCO2; best known as NDP52 (nuclear dot protein 52 kDa)) possesses a coiled-coil region, a noncanonical LIR, and a C-terminal UBZ domain [68]. Together with its close homologue Tax1bp1, NDP52 is localized to autophagosomes and subject to autophagic turnover [69,70]. NDP52 is recruited by intracellular Salmonella, binds ubiquitylated bacteria, and is an important xenophagy receptor. NDP52 can also be engaged indirectly via the intracellular lectin galectin 8 to interact with glycans and thereby participate in the clearance of protein aggregates [66].

While these receptors all mediate degradation of ubiquitylated cargos, other more specific adaptors act on removal of damaged or surplus organelles They recognize particular binding partners on the surface of their target organelle and, through their LIR sequence, ensure their delivery to the maturing autophagosome [71]. For example, mitophagy critically relies on BCL2 interacting protein 3-like (BNIP3L; best known as NIX) which requires LIR phosphorylation and receptor dimerization for proper BNIP3L-dependent mitophagy initiation and progression [72]. Upon induced expression or activation by hypoxia, these receptors localize to OMM and through their LIR motifs directly induce both formation of autophagosomes and mitophagy [73,74]. FUN14 domain containing 1 (FUNDC1), a protein of the outer mitochondrial membrane, also operates as autophagy receptor in response to hypoxia [73]. It contains three transmembrane domains and a cytosolic N-terminal part harboring a LIR that when phosphorylated at Tyr18 by Src regulates the interaction between FUNDC1 with LC3 [75]. TOLL-interacting protein (Tollip) also recruits and coordinates polyQ aggresome degradation via late endosome pathway in cellular models for Huntington’s disease, and it is considered more potent than p62 for polyQ aggresome clearance [76].

## 6. Signals That Determine Proteasomal and Lysosomal Degradation

The UPR regulatory proteins, PERK, IRE1α and ATF6α on the ER activate their own downstream pathways toward autophagy. The PERK-eIF2α sub-pathway facilitates the synthesis of the transcription factors ATF4 and CHOP, upregulating the expression of more than a dozen ATG genes [77] as well as LC3 lipidation and autophagosome biogenesis [78]. Meanwhile, activated IRE1 recruits TRAF2 to induce the phosphorylation of JNK. Phospho-JNK activates the expression of autophagic core genes via the XBP1s transcription factor and phosphorylates Bcl-2, leading to the dissociation of phospho-Bcl-2 from the PI3K class II complex and, thus, autophagosome biogenesis [79]. Finally, ATF6 migrates to and cleaved in the Golgi body and translocates to the nucleus, where it forms a heterodimeric transcription factor with C/EBP-β to induce the expression of DAPK1 and its phosphorylation of beclin-1 for autophagosome formation [80].

**Figure 4:**
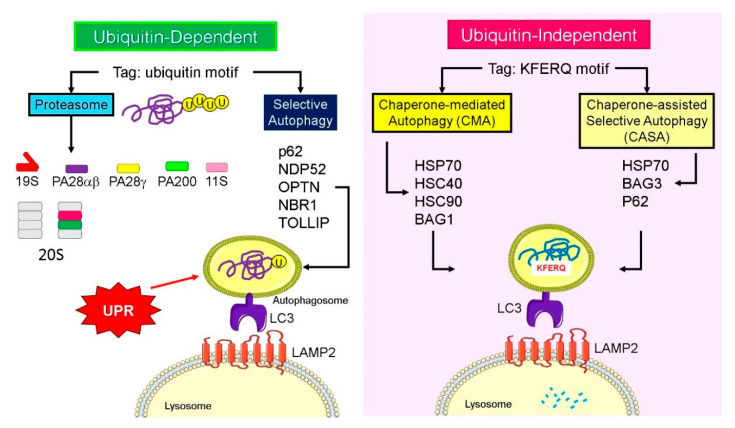
Adaptation for ubiquitin-dependent and -independent protein degradation. Combinations of proteasomal regulatory particles and autophagy adaptor proteins and possible heat-shock proteins and Bcl-2-associated athanogene proteins that allow adaptation of the protein degradation machinery depending on the cellular needs. Ubiquitylated proteins can be recognized various types of regulatory particles or by adaptive proteins that allow lysosomal degradation. Proteins with a specific recognition motif are either recognized by the chaperone-mediated (CMA) or chaperone-assisted selective (CASA) autophagy mechanisms to guide the cargo into the lysosomal degradation.

Cellular proteins containing a motif that are chemically related to the pentapeptide Lys-Phe-Glu-Arg-Gln (KFERQ) are recognized by cytoplasmic heat-shock chaperone HSC70 and its associated co-chaperones. While this chaperone-mediated autophagy (CMA) is ubiquitin-independent, lysosomes also have a ubiquitin-dependent selective system that is important during basal autophagy and starvation-induced autophagy (Figure 4). Histone deacetylase-6 (HDAC6) is a central component of basal autophagy, binds polyubiquitylated misfolded proteins and dynein motors, and by regulating microtubule acetylation loads misfolded proteins onto the microtubules for retrograde transport to the microtubule organizing center (MTOC) or aggresomes [81]. The cargo in turn assembles an F-actin cytoskeleton network that stimulates autophagosome–lysosome fusion [82]. SQSTM1/p62 and casein kinase II (CKII) increase the deacetylase activity by interacting with or phosphorylating HDAC6, respectively. Further, p97 [83], tripartite motif containing 50 (TRIM50), an E3 ubiquitin-ligase, and the cytokine-inducible ubiquitin-like modifier, FAT10/ubiquitin D, UBD, by associating with HDAC6, sequester polyubiquitylated proteins to the aggresome [84]. The distinct nature of basal autophagy is highlighted by the fact that HDAC6 and actin are dispensable for starvation-induced autophagy [82].

When the proteasomal function is inhibited and proteotoxic stress increases, Bcl-2-associated athanogene 3 (BAG3), is induced and interacts with HSP70 and with the dynein–dynactin motor complex to sequester proteins into aggresomes [1]. Therefore, Hsp70–Bag3 complex functions as an important signaling node that senses proteotoxicity and triggers protein aggregate formation [85]. Similarly, BAG-1 interacts with Hsc70 and Hsp70 chaperones, and functions as a link to the ubiquitin/proteasome system. Therefore, the BAG proteins, through their ubiquitin-like domain in the N-terminus, are considered sensors for enrichment of misfolded proteins and initiate the transport of aggregates to the lysosome for degradation (Figure 4). After interacting with the LAMP2A, the substrate is internalized through the membrane into the lysosomal lumen with the assistance of luminal HSC70 chaperone and rapidly degraded by luminal-resident proteases [86].

HSPB8 by interacting with BAG3 may define the assembly of primary deposit sites for ubiquitylated cytosolic proteins and facilitate their triaging for repair or degradation. HSPB8 and BAG3 appear to modulate both p62 phosphorylation and p62 levels, and thereby control the p62 sequestering activity [80]. Therefore, HSPB8 and BAG3 could be early sensors for switching the unfolded protein response proteasome pathway to autophagosome formation and lysosomal degradation.

## 7. Concluding Remarks

The cell has a highly balanced and targeted ubiquitylated system that tags misfolded proteins for elimination before they cause toxic damage to the complex network of processes that help survival. This review summarizes numerous studies that describe how the protein degradation machinery adapts to the needs of the cell. Depending on physiological state of the cell a complex proteasomal machinery that can adapt to cellular conditions by modifying its location and its regulatory units help to selectively attract the tagged proteins. The removed ubiquitin and the amino acids of degraded proteins are then recycled for new protein synthesis and antigen presentation. In addition, the UBL proteins together with scaffolding proteins and heat shock centers have evolved mechanisms to recognize larger degradation units, form double membranes that engulf selected aggregates for fusing with lysosomes to degrade and recycle the amino acid components. While both the proteasomal and lysosomal degradation systems work in tandem at baseline, a separate set of proteins signal either the assembly of specialized proteasomes or a set of adaptors activate the lysosomal degradation pathway to adapt to the need of the cell. Therefore, these systems fine-tune the speed of protein degradation depending on the environment, including pollutants that generate ROS or infectious agents that can initiate foreign protein synthesis. While components of the protein degradation system are somewhat understood, the sensors that allow speedy adaptation to support the innate and adaptive immunity of the organism are yet to be uncovered. Continued research effort in this area will help develop more effective treatment strategies for chronic inflammatory diseases and cancer.

## Figures and Tables

**Table 1:** Ubiquitinases and Deubiquitinases.

Ubiquitin Ligases	Number of Ligases	Deubiquitinases	Number of DUBs
Tag Proteins with Ubiquitin Residues		Remove a Ubiquitin Residue from Proteins.	
Ubiquitin activating enzyme E1 Ligase	2	Cystein Proteases: i. Ubiquitin specific proteases (USPs) ii. Ubiquitin carboxy-terminal hydrolases (UCHs) iii. Ovarian-tumor proteases (OTUs) iv. Machado-Joseph disease protein domain proteases (MJDs) v. Monocyte chemotactic protein-induced proteins (MCPIPs) vi. Permuted papain fold peptidases of dsRNA viruses and eukaryotes (PPPDEs)	62 USPs 4 UCHs 15 OTUs 4 MJDs 7 MCPIPs
Ubiquitin conjugating enzyme E2 Ligase	40	Zinc-dependent metalloproteinases: i. JAMMs/MPN+ proteases	4 JAMMs
Ubiquitin ligating enzymeE3 Ligase	600		

**Table 2:** Selective autophagy receptors.

Adapter Protein	Interaction Domain	Selective Autophagy Involved in
p62/SQSTM1	UBA	Aggrephagy, mitophagy, xenophagy, pexophagy and zymophagy
NBR1	UBA	Aggrephagy, mitophagy, xenophagy
Optineurin	UBAN, UBZ	Aggrephagy, mitophagy, xenophagy
NDP52	UBZ	Aggrephagy, mitophagy, xenophagy
BNIP3L/NIX	-	Mitophagy
FUNDC1	-	Mitophagy
HDAC6	-	Aggrephagy and mitophagy
